# Impact of Bariatric Surgery on Newborn Growth Parameters

**DOI:** 10.1155/ijpe/1763085

**Published:** 2026-04-30

**Authors:** Rayen Aoun, Souheil Hallit, Georges Nicolas

**Affiliations:** ^1^ School of Medicine and Medical Sciences, Holy Spirit University of Kaslik, Jounieh, Lebanon, usek.edu.lb; ^2^ Department of Cardiology, Notre Dame Des Secours University Hospital, Jbeil, Lebanon; ^3^ Applied Science Research Center, Applied Science Private University, Amman, Jordan, asu.edu.jo; ^4^ Department of Pediatrics, Notre Dame Des Secours University Hospital, Jbeil, Lebanon

**Keywords:** bariatric surgery, fetal development, nutritional deficiencies, obesity in pregnancy, pregnancy outcomes, small-for-gestational-age infants

## Abstract

**Objective:**

We are aimed at assessing the influence of different bariatric procedures on fetal growth and risk of small‐for‐gestational‐age (SGA) infants.

**Methods:**

This retrospective study was conducted at one hospital in Lebanon. A group of pregnant women with a history of bariatric procedures (*n* = 36) was compared with a control group of women without prior bariatric surgery (*n* = 98). Then, the study group was further stratified according to the type of procedure into purely restrictive and mixed bariatric techniques.

**Results:**

Pregnancies following bariatric surgery were associated with significantly lower mean birthweight, shorter birth length, and smaller head circumference, along with a decreased likelihood of large‐for‐gestational‐age infants. In contrast, the risk of SGA infants was higher.

**Conclusion:**

Our findings suggest a potential association between pregnancy following bariatric surgery and an increased risk of SGA infants. These results underscore the importance of providing appropriate preoperative counseling to women of reproductive age considering such procedures. For those with a history of bariatric surgery, optimizing nutritional status prior to conception and ensuring close monitoring of maternal nutrition and fetal growth throughout pregnancy are crucial. Preventive strategies aimed at reducing the risk of SGA outcomes in this population should be emphasized.

## 1. Introduction

Nowadays, obesity represents a major health issue in the world [1]. Its prevalence is rapidly growing worldwide, with recent data showing more than 650 million obese adults [2]. Although obesity is more common in developed countries, its frequency is also rising in developing countries due to the lifestyle changes and a propensity to follow an unhealthier diet [3].

It is well known that obesity increases the likelihood of developing several comorbidities, including diabetes mellitus, cardiovascular complications [4], and malignancy [5]. The prevalence of obese pregnant women has increased proportionally with the growing prevalence of obesity in the overall population [6]. Maternal obesity leads to a series of harmful complications for the mother and the newborn [7].

Recently, the clinical landscape for weight management has shifted toward the increasing use of glucagon‐like peptide‐1 (GLP‐1) receptor agonists as a main therapeutic approach for obesity [8]. In Lebanon, GLP‐1 receptor agonists, such as semaglutide and liraglutide, are increasingly prescribed for obesity management, reflecting a global trend in pharmacological weight control. However, there is a notable lack of large‐scale, specific studies conducted within the Lebanese population to assess long‐term effectiveness, safety, and patterns of cultural adherence specific to this population.

Therefore, bariatric surgery (BS) remains one of the most effective treatments for severe obesity [9]. It is commonly performed among women of reproductive age [10]. BS can be broadly classified into two main categories: restrictive procedures (e.g., sleeve gastrectomy) and malabsorptive procedures (e.g., gastric bypass) [1]. Patients who have undergone BS generally show a reduced risk of complications such as gestational diabetes and preeclampsia. Nevertheless, pregnancies following these procedures may still be associated with nutritional deficiencies [11].

Fetal growth is a crucial determinant of health. It can be affected by several interactions between genetic and environmental factors, including maternal nutrition [12]. Any mismatch between nutrients supplied by the placenta and the demand of the fetus can lead to abnormal fetal development. Birthweight has been widely used as a basic measure of intrauterine growth [13]. It also serves an important indicator in determining the infant’s growth and developmental outcomes [14].

BS can really have an impact on newborn growth parameters. Studies found that BSs may increase the likelihood of delivering small‐for‐gestational‐age infants [15]. Additionally, newborns of mothers with a history of BS had lower birth length, birthweight, and head circumference (HC) compared with newborns from mothers with no BS history [16].

We are conducting this study because, currently, the use of BS is rapidly increasing in Lebanon [17]. In fact, our country is witnessing nowadays an increased burden of obesity [18]. Unlike Western populations typically featured in large‐scale studies, the Lebanese demographic possesses a distinct genetic background and follows a Mediterranean‐based dietary pattern that is currently undergoing a “nutrition transition” toward more processed, calorie‐dense habits. Lebanese people tend to consume more fast foods and carbonated beverages and less dietary fibers [19]. In addition, it has been shown, in Lebanon, obesity is more prevalent among women compared with men (18.8% vs. 14.5%, respectively) [20]. Therefore, this study is aimed at evaluating fetal size at birth following BS, in order to better understand its impact on fetal health and determining whether it can negatively affect newborn growth parameters. Furthermore, this article compares the neonatal outcomes associated with restrictive versus malabsorptive procedures. The distinct physiological mechanisms of each type of these procedures can influence differently fetal growth and nutrient transfer. By including both types within our study, we provide a preliminary glimpse into how different surgical mechanisms may uniquely influence neonatal outcomes in a Middle Eastern context, thereby laying the groundwork for future, larger scale multicenter studies in the region. Hopefully, this study can help women of childbearing age and physicians assess carefully whether benefits outweigh potential adverse risks.

## 2. Methods

### 2.1. Study Design

This single‐center retrospective study was carried out using data obtained from the medical archives of Notre Dame des Secours University Hospital in Jbeil, Lebanon, between January 2013 and September 2021. Pregnant women were divided into two groups to assess the impact of BS on neonatal outcomes. The primary outcome was the occurrence of small‐for‐gestational‐age infants. Secondary outcomes included neonatal growth parameters (birthweight, birth length, and HC), occurrence of large‐for‐gestational‐age infants, preterm delivery, and Apgar scores. We included in the study group 36 pregnant women who had previously undergone BS, not necessarily at the same medical institution. The control group consisted of 98 overweight or obese pregnant women without a history of BS who delivered during the same period. A pregnancy in a woman without prior BS was included only once as a control. Multifetal pregnancies and aneuploidy were excluded due to their potential to affect fetal growth and increase complication rates.

### 2.2. Minimal Sample Size

Based on a previous study [16] showing a mean birthweight of 3284 ± 327 g in children of mothers who underwent BS versus 3619 ± 523 g in those whose mothers did not, the G‐power software estimated that at least 44 participants were required to achieve sufficient statistical power.

### 2.3. Data Collection Sheet

Fetal sex was recorded as male or female. Infants were classified as small (SGA), appropriate (AGA), or large for gestational age (LGA) based on Fenton growth charts. These categories were determined according to gestational age. SGA, AGA, and LGA were defined as a birthweight below the 10th percentile, between the 10th and 90th percentiles, and above the 90th percentile for gestational age, respectively.

Preterm birth was defined as delivery before 37 completed weeks of gestation [21]. Birthweight, birth length, and HC were recorded at the same time of delivery. Apgar scores were evaluated at 1 and 5 min after birth. Smoking status was categorized into two groups: women who had never smoked and those who smoked prior to pregnancy. Pre‐pregnancy body mass index (BMI) was determined as weight in kilograms divided by the square of height in meters [22].

Gestational weight gain (GWG) was computed as the difference between weight prior to delivery and pre‐pregnancy weight. Recommended GWG varies according to pre‐pregnancy BMI: 12.5–18 kg for underweight, 11.5–16 kg for normal weight, 7–11.5 kg for overweight, and 5–9 kg for obese women [23].

Parity was categorized as primiparous (no prior delivery) or multiparous (at least one prior delivery). Gestational age was estimated based on ultrasound findings.

Gestational diabetes mellitus (GDM) was diagnosed using an oral glucose tolerance test (OGTT), conducted between 24 and 28 weeks of gestation. Gestational hypertension was defined as newly developed hypertension (systolic blood pressure ≥ 140 mmHg or a diastolic blood pressure ≥ 90 mmHg) occurring after 20 weeks of gestation, without proteinuria or evidence of end‐organ damage [24]. Fetal delivery was classified as either vaginal or cesarean section. Maternal age was recorded at the same time of conception.

### 2.4. Statistical Analysis

Statistical analyses were conducted using SPSS Version 27. The chi‐square test was applied to compare categorical variables, whereas the independent samples *t*‐test was used for comparing means between two groups. A *p* value < 0.05 was considered statistically significant.

## 3. Results

Table 1 presents the main characteristics of the pregnant women included in the study. No significant differences were observed between the groups in terms of parity, GWG, chronic diseases, tobacco use, alcohol consumption, gestational hypertension, gestational diabetes, maternal age, and gestational age.

**Table 1:** Comparison between mothers who underwent bariatric surgery versus not in terms of sociodemographic and other characteristics.

Variable	No bariatric surgery (*N* = 98)	Bariatric surgery (*N* = 36)	*p*
Parity			0.618
Primipare	30 (30.6%)	15 (41.7%)	
Multipare	68 (69.4%)	21 (58.3%)	
BMI categories			**< 0.001**
Normal	0 (0%)	11 (30.6%)	
Overweight	40 (40.8%)	16 (44.4%)	
Obese	58 (59.2%)	9 (25.0%)	
Weight gain during pregnancy			0.581
Less than recommended	22 (22.7%)	10 (27.8%)	
Appropriate weight gain	22 (22.7%)	10 (27.8%)	
More than recommended	53 (54.6%)	16 (44.4%)	
Chronic diseases			0.620
No	83 (85.6%)	32 (88.9%)	
Yes	14 (14.4%)	4 (11.1%)	
Tobacco smoking			0.202
No	76 (77.6%)	24 (66.7%)	
Yes	22 (22.4%)	12 (33.3%)	
Alcohol drinking			0.337
No	92 (93.9%)	32 (88.9%)	
Yes	6 (6.1%)	4 (11.1%)	
Gestational hypertension			0.160
No	97 (99.0%)	34 (94.4%)	
Yes	1 (1.0%)	2 (5.6%)	
Gestational diabetes			0.570
No	93 (94.9%)	35 (97.2%)	
Yes	5 (5.1%)	1 (2.8%)	
Type of delivery			**0.032**
Normal	38 (38.8%)	21 (60.0%)	
Cesarean section	60 (61.2%)	14 (40.0%)	
Maternal age (in years)	31.74 ± 4.32	31.81 ± 4.04	0.941
Maternal BMI	31.04 ± 3.85	26.91 ± 3.80	**< 0.001**
Gestational age	37.82 ± 0.99	37.61 ± 1.52	0.361

*Note:* Numbers in bold indicate significant *p* values.

In Table 2, we focus on the outcomes of newborns in both groups. Post‐BS pregnancies, compared with nonbariatric control pregnancies, were associated with a significantly higher risk of SGA infants (13.9% vs. 0%; *p* < 0.001). Additionally, preterm birth rates were higher among women in the BS group (22.2% vs. 7.1%; *p* = 0.026). Moreover, infants in the BS group had significantly lower mean birthweight (2885.97 ± 432.44 g vs. 3244.69 ± 332.24 g; *p* < 0.001), shorter birth length (48.13 ± 1.56 cm vs. 48.93 ± 1.68 cm; *p* = 0.019), and smaller HC (33.91 ± 1.63 cm vs. 34.59 ± 1.26 cm; *p* = 0.017) were observed in infants born to mothers who underwent BS. Infants in the BS group showed a nonsignificant increase in Apgar scores at both 1 and 5 min.

**Table 2:** Comparison between mothers who underwent bariatric surgery versus not in terms of outcomes in the newborn babies.

Variable	No bariatric surgery (*N* = 98)	Bariatric surgery (*N* = 36)	*p*
Sex of the baby			0.577
Boy	57 (58.2%)	19 (52.8%)	
Girl	41 (41.8%)	17 (47.2%)	
Size of the baby			**< 0.001**
Small for gestational age	0 (0%)	5 (13.9%)	
Large for gestational age	7 (7.1%)	0 (0%)	
Appropriate for gestational age	91 (92.9%)	31 (86.1%)	
Prematurity			**0.019**
At term	91 (92.9%)	28 (77.8%)	
Preterm	7 (7.1%)	8 (22.2%)	
Birthweight (in g)	3244.69 ± 332.24	2885.97 ± 432.44	**< 0.001**
Birth length (in cm)	48.93 ± 1.68	48.13 ± 1.56	**0.019**
Head circumference (in cm)	34.59 ± 1.26	33.91 ± 1.63	**0.017**
Apgar at 1 min	8.35 ± 1.66	8.50 ± 1.25	0.615
Apgar at 5 min	9.27 ± 1.57	9.28 ± 1.14	0.965

*Note* :Numbers in bold indicate significant *p* values.

In Table 3, we compared the outcomes of infants born from mothers who underwent a restrictive BS (sleeve; LAGB) with newborns from mothers with a history of a malabsorptive procedure (RYGB). A significantly higher mean birth length was found in babies whose mothers underwent a sleeve/band type of surgery compared with those who did not, with this association tending to significance (48.43 ± 1.30 vs. 46.93 ± 2.01; *p* = 0.052). Nonsignificantly higher SGA rates were observed after RYGB.

**Table 3:** Comparison between the bariatric surgery types in terms of outcomes in the newborn babies.

Variable	Sleeve/band			Gastric bypass (RYGB)		
	No (*n* = 7)	Yes (*n* = 29)	*p*	No (*n* = 27)	Yes (*n* = 9)	*p*
Size of the baby			0.230			0.413
Small for gestational age	2 (28.6%)	3 (10.3%)		3 (11.1%)	2 (22.2%)	
Appropriate for gestational age	5 (71.4%)	26 (89.7%)		24 (88.9%)	7 (77.8%)	
Birthweight (in grams)	2646.43 ± 500.42	2943.79 ± 402.85	0.112	2937.41 ± 408.94	2731.67 ± 488.80	0.220
Birth length (in cm)	46.93 ± 2.01	48.43 ± 1.30	0.052	48.38 ± 1.31	47.39 ± 2.03	0.121
Head circumference (in cm)	33.00 ± 1.29	34.14 ± 1.65	0.102	34.19 ± 1.65	33.11 ± 1.36	0.093

## 4. Discussion

Our study shows a significantly higher SGA rate in pregnancies following BS than pregnancies without BS history. This is compatible with what was found with Johansson et al.; in their large Swedish study, they found that the risk of delivering SGA newborns following BS was approximately doubled (15.6% vs. 7.6%) compared with a matched control group without BS history [25]. Similarly, Getahun et al. observed an increased risk of SGA newborns in their BS group compared with the control group (16.6% vs. 8.3%) [11]. Infants born from mothers with BS history had a significantly lower birth length than babies in the control group. This was compatible with what was found in the study of Carlsen et al., where lower birth lengths were also found in the nonsurgical group [16]. Concerning HCs, newborns of mothers with BS history had a lower HC than infants in the control group (33.91 ± 1.63 cm vs. 34.59 ± 1.26 cm; *p* = 0.017). Similarly, Carlsen et al. reported lower HC in infants born to women with a BS history (34.2 vs. 35.1) [16]. The mechanisms underlying the impact of BS on birthweight and neonatal body composition are not completely understood but could be linked to altered intestinal nutrient absorption. In fact, the significant weight loss resulting from bariatric procedures may enhance insulin sensitivity and consequently reduce the availability of nutrients and substrates required for fetal growth. Another possible explanation is that nutrient and vitamin malabsorption, particularly after malabsorptive procedures, may impair GI absorption and decrease the placental transfer of nutrients from mother to fetus [11].

Bauer et al. conducted a study in which maternal intake was reduced for 20 days to levels below the recommended daily intake. They observed a decrease in insulin‐like growth factor‐1 levels (IGF‐1) [26]. IGF‐1 is a polypeptide hormone mainly synthesized in the liver and plays an essential role in fetal growth and development by regulating the actions of growth hormone. It has been reported in the literature that decreased levels of IGF‐I are predictive of impaired fetal development [27]. Additionally, experimental food restriction led to reduced placental weight and diminished placental efficiency [28]. Moreover, reduced placental weight may contribute to lower birthweight [29]. Studies have also shown a decrease in placental volume in cases of fetal growth restriction [30]. It is well established that restriction is a part of the physiological changes following bariatric procedures [31]. Therefore, it can be suggested that food restriction induced by BS may lead to decreased placental weight and function, ultimately resulting in abnormal fetal growth, low birthweight, reduced birth length, and smaller HC.

However, on the other hand, [32] contradicted this hypothesis and found no variations in SGA rates between BS patients and nonbariatric pregnant women [32]. However, that study included only pregnant women who underwent laparoscopic adjustable gastric banding, which is a purely restrictive technique, making it less likely to be associated with nutritional deficiencies. In addition, the sample size was relatively small compared with other studies.

Although SGA is a known correlate of immediate neonatal complications, the specific etiology of SGA matters when determining long‐term clinical outcomes. For example, SGA due to placental insufficiency, often characterized by restricted nutrient delivery and chronic hypoxia, presents a significantly different pathological profile than SGA in an otherwise healthy infant of a metabolically healthier mother, who may simply be constitutionally small. Distinguishing between these origins is vital for clinicians. Consequently, the clinical priority is not to discourage bariatric procedures in women of reproductive age, but rather to implement appropriate postsurgical nutritional management and specialized prenatal monitoring. By prioritizing the stabilization of maternal weight and the correction of micronutrient gaps prior to and during pregnancy, clinicians can preserve the significant long‐term metabolic benefits of the surgery while safeguarding fetal development.

On the other hand, significantly lower LGA rates were observed in the BS group compared with the control group (0% vs. 7.1%; *p* < 0.001). Chevrot et al. also reported a lower proportion of LGA infants in the bariatric group (11% vs. 22%) [1]. These findings may be explained by the lower prevalence of gestational diabetes and changes in nutritional status among patients who have undergone BS. Infants born LGA have higher odds for developing early‐onset obesity [33]. Therefore, reducing the incidence of LGA births may protect against the development of metabolic disease.

Although our study included a direct comparison between restrictive (sleeve gastrectomy) and malabsorptive (Roux‐en‐Y gastric bypass) procedures, we acknowledge that these subanalyses are likely underpowered due to the relatively small sample size in each surgical subgroup.

Research indicates that malabsorptive procedures are associated with a higher risk of small‐for‐gestational‐age infants [34]. This may be explained by the mechanisms of malabsorption, which lead to increased rates of nutritional deficiencies during pregnancy. However, in our study, there were more cases of sleeve than Roux‐en‐Y gastric bypass, which limited the ability to perform a better comparison of the influence of different types of BS.

In addition, our study shows higher rates of preterm births in the surgical group compared with the control group (22.2% vs. 7.1%; *p* = 0.026). This is consistent with findings from the large Swedish study of Roos et al. [15] This can be explained by the role of nutritional deficiencies in contributing to several mechanisms involved in preterm delivery (e.g., infection, inflammation, oxidative stress, and muscle contractility) [35]. Malabsorptive procedures (such as Roux‐en‐Y gastric bypass) may pose a greater risk than restrictive ones, as they bypass segments of the small intestine where essential nutrients are absorbed.

Pregnant women in both groups were almost matched by their maternal age, which decreases the possible effects of aging. Advanced maternal age is associated with reduced fetal growth potential, possibly reflecting age‐related changes in maternal physiological systems [36]. Furthermore, older mothers tend to have a higher likelihood of chronic conditions and pregnancy‐related complications, including obesity, anemia, and diabetes [37].

All bariatric pregnant women included in our study delivered at least 1 year after undergoing BS, with an average interval of 4.39 years. Evidence from prior research suggests that the likelihood of adverse neonatal outcomes following BS is greater when conception occurs within the first year after surgery. This interval is characterized by significant caloric restriction and rapid weight loss, which may predispose mothers to nutritional deficiencies that could compromise fetal nutrient supply [38]. Kjær et al. compared pregnancy outcomes within 12 months and after 1 year of BS and reported a nonsignificant increase in adverse perinatal outcomes [39]. Heusschen et al. found that birthweight was lower by approximately 200 g in pregnancies within 1 year [40]. However, Getahun et al. reported that the association between BS and increased risk of SGA offspring was independent of the time interval between surgery and conception [11].

Our institution follows a comprehensive multidisciplinary protocol for infants born to mothers with a history of BS, ensuring a holistic approach to postnatal care. This specialized care team—composed of pediatricians, clinical nutritionists, and neonatologists—closely monitors infants to reduce potential risks such as intrauterine growth restriction or micronutrient deficiencies. Follow‐up begins at birth with detailed anthropometric assessments (including weight, length, and HC) and continues at regular intervals to monitor growth patterns and developmental milestones.

## 5. Strengths and Limitations

One of the strengths of our study is that it was not limited to a single bariatric procedure; it included both restrictive and malabsorptive techniques. Additionally, the women included in the surgical group were all pregnant at least 12 months after surgery, which may reduce the influence of rapid weight loss occurring during the first postoperative year.

One of the study limitations is that data were obtained from a single center, which may introduce selection bias, as the sample may not fully represent the general population. In addition, the relatively small sample size of BS patients included from our center can be explained by the tendency of patients to initially opt for pharmacological treatments before considering surgical options. Also, external barriers play a significant role, including limited insurance coverage. Additionally, data on the nutritional status of pregnant women during pregnancy were not available, which did not allow us to check if participants were following their dietary recommendations.

## 6. Conclusion

In summary, our study highlights a potential association between pregnancy after BS and a higher risk of SGA infants. These findings could contribute to better informed counseling of women of reproductive age before undergoing such procedures. For women with a history of BS, improving nutritional status prior to conception and ensuring careful monitoring of fetal growth and maternal nutrition during pregnancy are essential. Preventive strategies aimed at reducing SGA outcomes in this population should be emphasized. Further studies are needed to explore the extent to which appropriate nutritional management following BS can help prevent adverse fetal growth outcomes.

## Author Contributions

G.N. was involved in the conceptualization of the study. R.A. curated the data. Data collection was conducted by R.A. and G.N. The formal analysis was carried out by S.H. Methodology was developed by R.A., G.N., and S.H. G.N. and S.H. provided supervision. The original draft of the manuscript was written by R.A. The manuscript was reviewed and edited by S.H. and G.N.

## Funding

No funding was received for this manuscript.

## Disclosure

All authors read and approved the final manuscript. Our abstract was approved to be presented online as a poster in a conference of the European society for pediatric endocrinology. The name of the conference is the 60th Annual ESPE Meeting 2022. The theme of the conference is the personalized medicine in pediatric endocrinology. This conference took place on September 15–17, 2022. This conference was held at Roma, Italy.

## Ethics Statement

Before starting data collection, this study was approved by the ethical committee of the Notre Dame des Secours University Hospital Center, Jbeil. Patient′s informed consent was not taken because our study is retrospective, and data were collected from the archives (which was approved by the Ethics Committee).

## Consent

The authors have nothing to report.

## Conflicts of Interest

The authors declare no conflicts of interest.

## Data Availability

The data that support the findings of this study are available on request from the corresponding author. The data are not publicly available due to privacy or ethical restrictions.
